# SGPP2 Ameliorates Chronic Heart Failure by Attenuating ERS via the SIRT1/AMPK Pathway

**DOI:** 10.3390/cimb48010100

**Published:** 2026-01-19

**Authors:** Yang Kang, Yang Wang, Lili Wang, Lu Fu

**Affiliations:** 1 Department of Cardiology, The First Affiliated Hospital of Harbin Medical University, No. 199 Dongdazhi Street, Nangang District, Harbin 150001, China; 2 The First Clinical Medical College of Harbin Medical University, No. 199 Dongdazhi Street, Nangang District, Harbin 150001, China

**Keywords:** chronic heart failure, endoplasmic reticulum stress, SGPP2, SIRT1/AMPK

## Abstract

**Objective:** To investigate the mechanism by which sphingosine-1-phosphatase 2 (SGPP2) modulates endoplasmic reticulum stress (ERS) through the SIRT1/AMPK pathway to improve ischemic cardiomyopathy-induced chronic heart failure (IHF). **Methods:** Key genes of IHF and ERS were identified through bioinformatics analysis, and significantly associated pathways of the key genes were obtained via single-gene enrichment analysis. In vivo, IHF was induced in Sprague–Dawley (male) rats via ligation of the left anterior descending coronary artery, with cardiac function examined through echocardiography. Myocardial tissue injury and fibrosis were evaluated utilizing hematoxylin-eosin, Masson, and TUNEL staining. Serum levels of NT-proBNP and cTnT were measured via ELISA. SGPP2 protein expression was assessed via immunohistochemistry and Western blotting (WB). In vitro, neonatal rat cardiomyocytes (NRCMs) were isolated and underwent oxygen-glucose deprivation (OGD) to establish an IHF model. SGPP2-overexpressing NRCMs were constructed and treated with the ERS inducer tunicamycin (Tu) or the SIRT1 inhibitor EX527. Cell injury was evaluated using Cell Counting Kit-8 and lactate dehydrogenase release assays, as well as flow cytometry. Endoplasmic reticulum structure was examined by transmission electron microscopy. The endoplasmic reticulum was labeled with the ER-Tracker Red molecular probe. WB was utilized to detect the expression of apoptosis- and ERS-linked proteins, and the activity of the SIRT1/AMPK signaling pathway. **Results:** Six key genes (CTSK, FURIN, SLC2A1, RSAD2, SGPP2, and STAT3) were identified through bioinformatics analysis, with SGPP2 showing the most significant differential expression. Additionally, SGPP2 was found to be downregulated in IHF. Single-gene enrichment analysis showed that SGPP2 exhibited a significant association with the AMPK signaling pathway. Animal experiments demonstrated that rats with IHF exhibited significantly impaired cardiac function, marked myocardial tissue injury and fibrosis, ERS in myocardial tissue, lowered SGPP2 expression, and decreased SIRT1/AMPK signaling pathway activity. In vitro experiments confirmed that SGPP2 overexpression alleviated OGD-induced cardiomyocyte injury by inhibiting ERS and simultaneously activating the SIRT1/AMPK signaling pathway. Rescue experiments further demonstrated that both Tu and EX527 significantly promoted ERS and cellular injury, thereby counteracting the protective effects of SGPP2. **Conclusions:** SGPP2 alleviates IHF by inhibiting ERS modulated by the SIRT1/AMPK pathway.

## 1. Introduction

Heart failure (HF), a heterogeneous clinical syndrome, notably features high morbidity and death rates [1]. Its predominant etiologies encompass ischemic cardiomyopathy (ICM) and dilated cardiomyopathy. The former can lead to cardiomyocyte injury, death, and ventricular remodeling. This process forms a progressive vicious cycle, ultimately resulting in ICM-induced chronic HF (IHF) [2]. Endoplasmic reticulum stress (ERS) significantly influences the progression of multiple cardiovascular diseases and is a recognized key pathophysiological process in HF development and progression [3]. Accumulating evidence indicates that ERS promotes cardiomyocyte apoptosis in myocardial infarction [4].

The endoplasmic reticulum is a highly dynamic organelle. Its functional homeostasis can be disrupted by various stimuli, causing the accumulation of unfolded or misfolded proteins in its lumen, a process referred to as ERS [5]. In the early stages, ERS primarily alleviates misfolded protein accumulation and attempts to restore endoplasmic reticulum homeostasis by activating the unfolded protein response (UPR) [5]. UPR involves three signaling pathways activated by endoplasmic reticulum-resident proteins: the protein kinase R-like endoplasmic reticulum kinase (PERK), the inositol-requiring protein 1, and the activating transcription factor (ATF) 6 signaling pathways [6]. Under conditions of severe ERS, increased ATF4 enters the nucleus and interacts with C/EBP-homologous protein (CHOP) for apoptosis-linked gene expression regulation, thereby promoting cell death.

Activating the AMP-activated protein kinase (AMPK) signaling pathway regulates energy supplementation and reduces inflammation, thereby conferring cardiovascular protection in patients with HF [7]. Furthermore, AMPK alleviates alcohol-induced myocardial injury by modulating ERS [8]. Quercetin alleviates sepsis-induced acute lung injury by activating the sirtuin 1 (SIRT1)/AMPK pathway to suppress oxidative stress (OS)-mediated ERS [9]. Similarly, sitagliptin alleviates α-naphthyl isothiocyanate-triggered cholestatic liver injury by targeting SIRT1/AMPK to protect against OS-regulated ERS and inflammation [10]. Collectively, these studies suggest that regulating ERS by targeting the SIRT1/AMPK signaling pathway is promising in treating cardiovascular diseases like HF.

Sphingosine-1-phosphatase 2 (SGPP2) is a transmembrane protein that dephosphorylates sphingosine-1-phosphate (S1P). A transcriptomic and metabolomic analysis has revealed the low expression of SGPP2 in the myocardial tissue of patients with tetralogy of Fallot [11]. Consistently, in an animal study, cells from SGPP2^−/−^ mice were demonstrated to exhibit significantly increased expression of ERS characteristic proteins compared to those from wild-type mice [12]. Therefore, SGPP2 may significantly mediate cardiovascular disease and ERS. Nevertheless, its function and underlying molecular mechanisms in HF remain elusive. Therefore, the present study endeavors to address this gap, with an attempt to build a theoretical basis and present novel insights for targeted therapeutic strategies to ameliorate clinical outcomes in the HF population.

## 2. Materials and Methods

### 2.1. Differential Gene Expression Analysis

To investigate the association between ERS and IHF, we performed a series of bioinformatic analyses. GSE57338 (comprising 95 IHF and 136 normal samples) and GSE79962 (comprising 11 IHF and 11 normal samples) were downloaded from the GEO database using the GEOquery package (v 2.68.0). Differential expression analysis (DEA) of GSE57338 was conducted via “limma” (v 3.56.2), and genes meeting the criteria of *p* < 0.05 and |log_2_FC| > 0.5 were defined as differentially expressed genes (DEGs). ERS-related genes were collected through a comprehensive literature review in the PubMed database. Gene set variation analysis (GSVA) was subsequently carried out to derive GSVA enrichment scores, and the differences in ERS-related GSVA scores between IHF and normal samples were statistically evaluated.

### 2.2. Weighted Gene Co-Expression Network Analysis (WGCNA)

A network modularity analysis was performed on GSE57338 via “WGCNA” (v 1.72.1). Hierarchical clustering of samples was conducted with the stats package (v 4.3.1) based on the mean, and outlier samples were removed. Subsequently, a soft threshold of 7 was selected to ensure that the constructed network could better conform to the characteristics of a scale-free network. Module clustering analysis was performed on all genes, and Pearson correlation analysis of different clustering modules was performed through “base” (v 4.3.1). Based on the screening criterion of a correlation greater than 0.5, genes from modules significantly correlated with IHF were identified. These module genes were then intersected with IHF-related DEGs and ERS-related genes to obtain overlapping genes. Finally, a heatmap of the overlapping genes was plotted using the pheatmap package (v 1.0.13).

### 2.3. Functional Enrichment Analysis (FEA) and Protein–Protein Interaction (PPI) Analysis

FEA of the overlapping genes was enabled utilizing DAVID (https://davidbioinformatics.nih.gov/, 5 May 2023). The top 10 terms from GO and KEGG pathway enrichment analyses were derived. PPI analysis of the overlapping genes was carried out using STRING (https://cn.string-db.org/, 5 May 2023) with a confidence threshold of 0.4.

### 2.4. Machine Learning (ML) Analysis

The overlapping genes were analyzed, and key candidate genes were found via ML approaches. The following three algorithms were applied: (1) random forest analysis utilizing “randomForest” (v 4.7.1.1); (2) SVM-RFE analysis employing “e1071” (v 1.7.13); and (3) LASSO-cox analysis using “glmnet” (v 4.1.8). The gene sets from all three algorithms were intersected to obtain the final set of key genes. The expression levels of these key genes were compared across IHF and normal samples using the training set GSE57338 and the validation set GSE79962. The final results were visualized via “ggplot2” (v 3.4.1).

### 2.5. Single-Cell Analysis

GSE145154 (comprising 4 normal samples and 6 IHF samples) was obtained using the GEOquery package (v 2.68.0). Single-cell analysis of the GSE145154 dataset was performed using the Seurat package (v 4.4.0). Cell annotation was conducted using the SingleR package (v 2.2.0). Differential expression of the key genes was evaluated across different cell types between IHF and normal samples, and the results were visualized using the ggplot2 package.

### 2.6. Immune Infiltration Analysis

CIBERSORTx (https://cibersortx.stanford.edu/runcibersortx.php) (10 May 2023) was used to perform CIBERSORT analysis on the GSE57338 dataset. First, the expression values and proportions of different cell types in each sample were quantified. Differences in immune infiltration scores between the IHF and normal groups were subsequently assessed for each cell type. Pearson correlation analysis was carried out to unveil correlations among significantly different cell types, as well as between key genes and these significantly different cell types. All results were visualized via “ggplot”.

### 2.7. Single-Gene Enrichment Analysis (SGEA)

SGEA of key genes was performed. The top 200 genes most significantly co-expressed with the key genes were obtained from GTEx data utilizing “clusterProfiler” (v 4.8.3). Gene symbols were converted to Entrez IDs, and KEGG analysis was performed using “enrichKEGG” in clusterProfile (v 4.8.3) (*p*-value < 0.05). The pathway exhibiting the lowest P-value was subsequently visualized.

### 2.8. Animal Model and Surgical Procedures

Sprague–Dawley (SD) rats (Male, 200-250 g) (*n* = 14) were housed in the animal facility of the First Affiliated Hospital of Harbin Medical University. IHF was triggered by ligation of the left anterior descending coronary artery to simulate ICM (*n* = 8). Rats, anesthetized with Zoletil-50, were put in a supine position on a dissection table. The surgical area was shaved, prepared, and disinfected. Tracheal intubation was performed and the tube was connected to a small animal ventilator. Ribs were separated to expose the heart, and the coronary artery was ligated 2-3 mm below the left auricle utilizing a surgical suture. Successful ligation was confirmed by electrocardiographic changes, including ST-segment elevation, and immediate pallor of the ligated area. Sham-operated rats (*n* = 6) underwent the identical process without ligation. One week following surgery, echocardiography was carried out, and a left ventricular ejection fraction (LVEF) < 50% denoted successful model induction. Histopathological examinations were conducted four weeks later. All animal experiments were approved by the Institutional Animal Care and Use Committee of the First Affiliated Hospital of Harbin Medical University (approval code: 2024095; approval date: 20 March 2024).

### 2.9. Echocardiographic Examination

Rats, anesthetized with Zoletil-50, were positioned in a supine posture on a pre-warmed platform, with the body securely fixed. Following hair removal, the skin was disinfected with alcohol. Subcutaneous ECG needle electrodes were inserted into the forelimbs and/or hindlimbs and connected to leads. M-mode images were obtained at the papillary muscle level in the short-axis view. Left ventricular end-diastolic diameter (LVEDD) and left ventricular end-systolic diameter (LVEDS) were derived, and LVEF and left ventricular fractional shortening (LVFS) were subsequently calculated.

### 2.10. Measurement of Serum NT-proBNP and cTnT

Blood was drawn from the abdominal aorta of rats under anesthesia. Serum levels of NT-proBNP and cTnT were measured utilizing ElaBoX™ Rat NT-proBNP ELISA Kit (SEKR-0097, Solarbio, Beijing, China) and ElaBoX™ Rat cTnT ELISA Kit (SEKR-0047, Solarbio). Optical density (OD) was derived via a microplate reader (MK-3, Thermo Fisher Scientific, Waltham, MA, USA).

### 2.11. Hematoxylin-Eosin (HE) Staining

Cardiac tissues were harvested immediately after blood collection and fixed for 24 h. Subsequently, they were cut into slices (4–6 μm) according to the standard protocol. HE staining was performed following the instructions of the HE Stain Solution (G1125, Solarbio). After mounting, the foregoing sections were observed and imaged under a light microscope (Primo Star, Zeiss, Jena, Germany).

### 2.12. Masson Staining

Myocardial fibrosis was examined via a Masson’s trichrome staining kit (G1340, Solarbio). Deparaffinized and rehydrated tissue sections were stained with Ponceau S-Fuchsin (10 min), subjected to phosphomolybdic acid (2 min), and stained with aniline blue (2 min), with weak acid rinses performed between each step. Sections were fixed after dehydration and clearing, and imaged under an optical microscope.

### 2.13. Detection of Apoptosis in Tissue Sections

After deparaffinization and rehydration, the antigen was retrieved utilizing citrate buffer with microwave heating. After natural cooling, the tissue sections were washed (three times, for 5 min each). TUNEL staining was performed using the TUNEL Cell Apoptosis Detection Kit (DAB Method) (G4891, Solarbio). After PBS washes, the sections were dried, mounted, and imaged.

### 2.14. Immunohistochemistry (IHC)

Paraffin-embedded tissue sections were treated following the protocol described for TUNEL staining and washed with PBS three times. Blocking was conducted using normal goat serum for 30 min at room temperature. Sections were incubated at 4 °C in a humid chamber overnight with anti-SGPP2 antibody (5 µg/mL, LS-C448026, UnivBio, Shanghai, China). After PBS washes, the sections were incubated with goat anti-rabbit IgG-HRP for 60 min at room temperature before additional PBS washes. DAB staining was carried out before hematoxylin counterstaining. Sections were fixed after dehydration and clearing, and imaged.

### 2.15. Cell Culture and Treatment

Neonatal rat cardiomyocytes (NRCMs) were isolated from postnatal day 1–2 SD rats. Briefly, the neonatal rats were exposed to low temperature until unresponsive and then euthanized by cervical dislocation. Cardiac tissues were collected, washed with D-Hank’s solution, and digested with 0.075% trypsin and 0.1% collagenase type II. Cells were suspended in high-glucose Dulbecco’s modified Eagle medium (DMEM) (11965092, Gibco™, Grand Island, NY, USA) with 100 U/mL penicillin, 100 mg/mL streptomycin, and 10% fetal bovine serum (A5256701, Gibco™), and then cultured for 1.5 h at 37 °C in a humidified environment with 5% CO_2_ [13]. NRCMs were seeded into plates with 24 wells and maintained at 37 °C under 5% CO_2_ in humidified air. The identity of NRCMs was confirmed by immunofluorescence staining for α-actinin with DAPI counterstaining (Supplementary Figure S1).

NRCMs were subjected to oxygen-glucose deprivation (OGD) [14]. They were cultured in serum- and glucose-free DMEM and placed in a tri-gas incubator supplied with 95% N_2_, 5% CO_2_, and 1% O_2_ for hypoxia. Before OGD, the DMEM medium was pre-equilibrated under hypoxic conditions for 30 min to reduce its oxygen content.

SGPP2 overexpression plasmid (oe-SGPP2) and its negative control plasmid (oe-NC) (GenePharma, Shanghai, China) were transfected into NRCMs using Lipofectamine 3000 reagent (L3000075, Invitrogen, Carlsbad, CA, USA) to achieve SGPP2 overexpression. During OGD induction, the ERS inducer tunicamycin (Tu, 10 µM) was added to the culture medium of SGPP2-overexpressing NRCMs. Additionally, before OGD induction, SGPP2-overexpressing NRCMs were pretreated for 2 h with the SIRT1 inhibitor EX527 (HY-15452, MCE, Merced, CA, USA).

### 2.16. Cell Counting Kit-8 (CCK-8) Assay

NRCMs were seeded into the pre-incubated 96-well plates and cultured under specified conditions. Following experimental treatments, 10 µL of CCK-8 solution (C0037, Beyotime, Shanghai, China) was added, and bubble formation was prevented. Plates were returned to the incubator and incubated for 1 h. Subsequently, the OD was measured via a microplate reader.

### 2.17. Lactate Dehydrogenase (LDH) Release Assay

LDH release was measured via WST-8 (C0019S, Beyotime). Briefly, cell culture supernatants were transferred to a 96-well plate, with 100 µL per well. Then, 100 µL of the LDH detection working solution was added. The reaction was terminated after 10 min, and the OD at 450 nm was measured using a microplate reader.

### 2.18. Flow Cytometry

The 100,000 cells were resuspended in 195 μL Annexin V-FITC binding buffer (C1062L, Beyotime) before staining with 5 μL Annexin V-FITC and 10 μL propidium iodide. After 15 min, samples were analyzed via flow cytometry immediately to derive the apoptosis rate (%).

### 2.19. Transmission Electron Microscopy (TEM)

For myocardial tissue, samples were rapidly harvested and immediately fixed with pre-cooled glutaraldehyde, followed by post-fixation with osmium tetroxide. Tissues were dehydrated through a graded ethanol series, infiltrated, and embedded in epoxy resin, which was polymerized by heating at 60 °C. An ultramicrotome was utilized to prepare ultrathin sections (50 nm), which were then collected on copper grids and stained sequentially with uranyl acetate and lead citrate. The endoplasmic reticulum was ultimately observed under a transmission electron microscope (H-7650, Hitachi, Tokyo, Japan).

### 2.20. ER-Tracker Red Staining

NRCMs were cultured in 6-well plates until cell density reached 50~70%. Then, cells were washed twice using serum-free medium. ER-Tracker Red (1 μM, E34250, Invitrogen) was added. Cells were incubated in the dark for 30 min at 37 °C with 5% CO_2_. After incubation, the staining solution was discarded, and the cells were rinsed again with serum-free medium. Fresh pre-warmed complete medium was then added, and imaging was performed immediately using a fluorescence microscope (Axio Scope A1, Zeiss, Germany).

### 2.21. Western Blot (WB)

Total protein was extracted utilizing RIPA lysis buffer (P0013B, Beyotime) containing 1% PMSF. Protein concentration was determined via BCA (P0010, Beyotime). Protein samples were loaded onto 10% SDS-PAGE gels (P0012A, Beyotime) and subsequently transferred to PVDF membranes. The membranes were incubated with the following primary antibodies: Caspase3 (casp3) antibody (#9662, 1/1000), Cleaved-casp3 (#9661, 1/1000), PERK antibody (#3192, 1/1000), p-PERK antibody (#3179, 1/1000), eIF2α antibody (ab169528, 1/1000), p-eIF2α antibody (68023-1-Ig, 1/5000), glucose-regulated protein (GRP)-78 antibody (ab21685, 1/1000), ATF4 antibody (ab216839, 1/1000), CHOP antibody (66741-1-Ig, 1/1000), SGPP2 antibody (2 µg/mL), SIRT1 antibody (#9475, 1/2000), AMPK antibody (#2532, 1/1000), p-AMPK antibody (#2535, 1/1000), and β-actin (#4967, 1/1000). Afterwards, the membranes were incubated with an anti-rabbit IgG (HRP) (ab6721, 1/5000). Target proteins were visualized. All antibodies mentioned above (except SGPP2) were bought from Proteintech Group, Inc. (Rosemont, IL, USA), Cell Signaling Technology, Inc. (Danvers, MA, USA), or Abcam (Shanghai) Trading Co., Ltd. (Shanghai, China).

### 2.22. Statistical Analysis

The statistical data are shown in mean ± standard deviation. All data meet the test of normality and homogeneity of variance. Differences across groups were analyzed utilizing Student’s *t*-test, while comparisons across groups were carried out via one-way ANOVA. Statistical analyses were performed in GraphPad Prism (v 8.0). *p* < 0.05 signified statistical significance.

## 3. Results

### 3.1. DEA of IHF-Related Genes and Analysis of ERS-Related Genes

**Figure 1:**
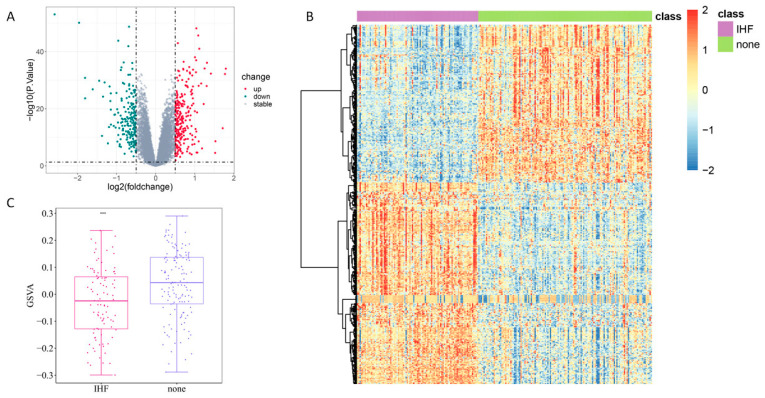
DEA of IHF-related genes and analysis of ERS-related genes. (**A**): Volcano plot displaying the distribution of DEGs. (**B**): Heatmap illustrating the expression patterns of DEGs across groups. (**C**): ERS-associated genes. *** *p* < 0.001 vs. the 0.001 vs. the none group.

DEA of genes in the GSE57338 dataset revealed 249 up-regulated genes and 195 down-regulated genes (Figure 1A). A heatmap presenting the expression patterns of these DEGs is provided in Figure 1B. Moreover, 785 ERS-related genes were retrieved. As shown in Figure 1C, the expression of ERS-related genes was notably down-regulated in the IHF cohort relative to the none group.

### 3.2. WGCNA

**Figure 2:**
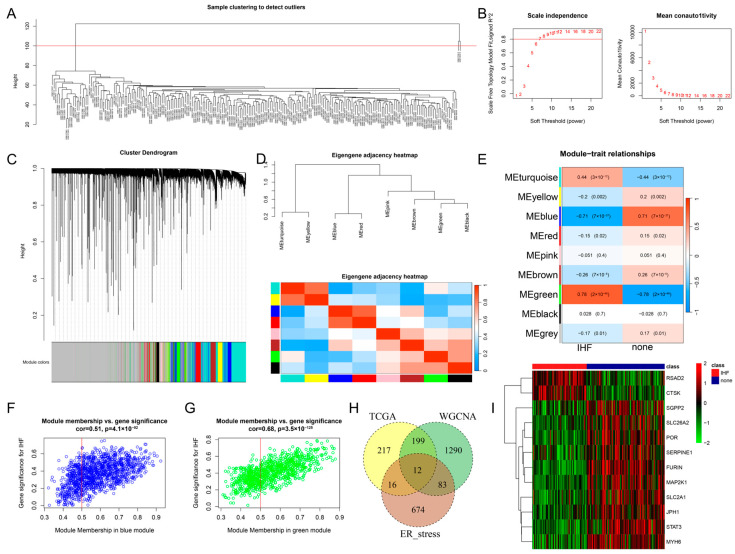
WGCNA. (**A**): Hierarchical clustering. (**B**): Topological analysis and average connectivity analysis. (**C**): Module clustering analysis. (**D**): Module correlation analysis. (**E**): Module-disease correlation analysis. (**F**,**G**): Analysis of correlation of module membership with gene significance. (**H**): Venn diagram displaying the overlapping genes among IHF-linked DEGs and ERS-linked genes. (**I**): Correlation analysis of the overlapping genes.

Following hierarchical clustering, two samples that were markedly distinct from the others were removed (Figure 2A). As shown in Figure 2B, a soft threshold of 7 was chosen as per the criterion of achieving a scale-free topology fit index above 0.8 (left panel), under which the decline curve of mean connectivity reached a stable plateau (right panel). The cluster dendrogram illustrates the hierarchical structure of the data, enabling the identification of inherent clustering patterns (Figure 2C). The cluster dendrogram and module correlation heatmap are presented in Figure 2D. Figure 2E further illustrates the correlation between modules and IHF/none. Based on a correlation threshold > 0.5, two modules, blue (1001 genes, Figure 2F) and green (583 genes, Figure 2G), were identified. Subsequently, the 1584 genes from these modules were intersected with the DEGs and ERS-related genes, resulting in the identification of 12 overlapping genes (Figure 2H): RSAD2, CTSK, SGPP2, SLC26A2, POR, SERPINE1, FURIN, MAP2K1, SLC2A1, JPH1, STAT3, and MYH6. Finally, the expression patterns of these 12 overlapping genes were visualized using a heatmap generated by the pheatmap package (Figure 2I).

### 3.3. FEA and PPI Analysis

**Figure 3:**
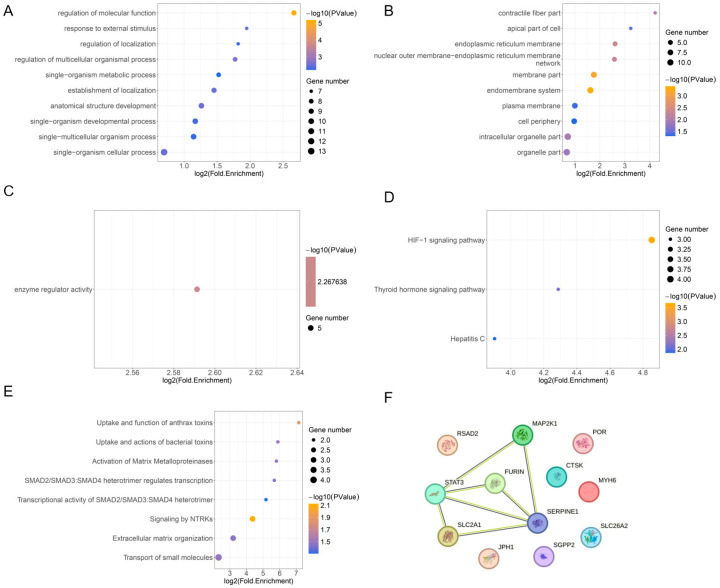
FEA and PPI analysis. (**A**–**C**): Top 10 GO-BP, GO-CC, and GO-MF terms. (**D**): KEGG pathways. (**E**): Reactome pathways. (**F**): PPI network.

The enrichment analysis results of the 12 overlapping genes are summarized in Supplementary File S1. The top 10 GO-BP, GO-CC, and GO-MF terms are shown in Figure 3A–C. GO enrichment analysis indicated the primary involvement of overlapping genes in responses to external stimuli, regulation of molecular functions, and modulation of cellular localization, while also being implicated in developmental and metabolic processes. KEGG pathway enrichment analysis (Figure 2D) suggested that these genes are mainly associated with hypoxia stress, metabolic reprogramming, and viral infection/inflammatory responses. Reactome pathway enrichment results (Figure 3E) revealed that the biological functions of these genes primarily involve cellular endocytosis of toxins/molecules, TGF-β signaling activation, extracellular matrix degradation and reorganization, and potential neurotrophic signaling. Therefore, the overlapping genes may regulate cancer metastasis, tissue fibrosis, and embryonic development. The PPI network (Figure 3F) showed limited interactions among the 12 overlapping genes.

### 3.4. ML Analysis of Core Genes

**Figure 4:**
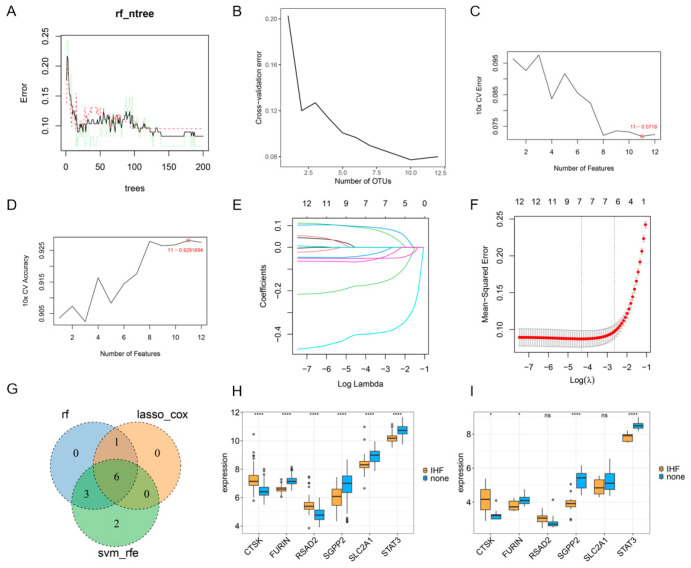
ML analysis and core gene validation. (**A**,**B**): Random forest analysis. (**C**,**D**): SVM-RFE analysis. (**E**,**F**): Coefficient path diagram of Lasso-Cox regression. (**G**): Six key genes screened by three algorithms. (**H**,**I**): Expression validation of key genes. The lines of different colors in Figure **E** represent different variables. * *p* < 0.05, **** *p* < 0.0001, ns represents unlimited statistical difference.

10, 11, and 7 genes were identified through random forest analysis (Figure 4A,B), SVM-RFE analysis (Figure 4C,D), and the Lasso-Cox model (using lambda 1se) (Figure 4E,F), respectively. The intersection of the results from all three ML algorithms resulted in the identification of six key genes: CTSK, FURIN, SLC2A1, RSAD2, SGPP2, and STAT3 (Figure 4G). Among these key genes, FURIN, SGPP2, SLC2A1, and STAT3 were expressed at low levels in the IHF cohort, while CTSK and RSAD2 were expressed at high levels (Figure 4G). Validation analysis (Figure 4I) confirmed that SGPP2, FURIN, and STAT3 were downregulated in the IHF group, whereas CTSK was upregulated, with SGPP2 showing the most prominent differential expression.

### 3.5. The Results of Single-Cell Analysis

**Figure 5:**
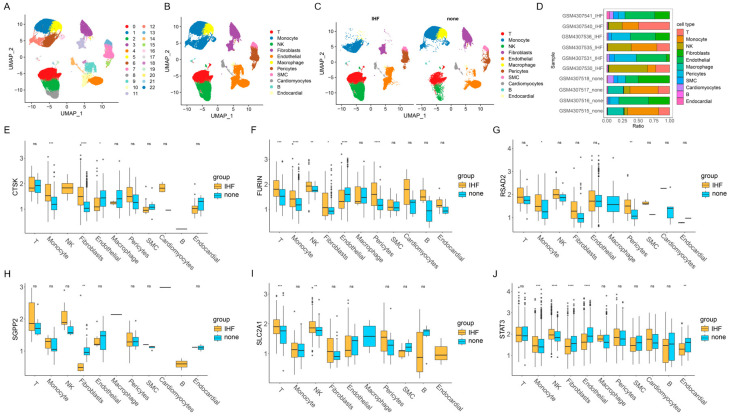
Single-cell analysis. (**A**): UMAP distribution of cell clustering. (**B**): UMAP distribution of cell type annotation. (**C**): Comparison of UMAP distribution of cell type annotation across cohorts. (**D**): Proportion of different cell types in each sample. (**E**–**J**): Differences in SGPP2 expression across cell types across groups. * *p* < 0.05, ** *p* < 0.01, *** *p* < 0.001, **** *p* < 0.0001, ns represents unlimited statistical difference.

23 cell clusters were identified through cell clustering of the GSE145154 dataset (Figure 5A). Subsequent cell-type annotation (Figure 5B) revealed 11 cell types, primarily comprising abundant structural/stromal cells and a large number of immune cells. The distribution of different cell types varied significantly across groups (Figure 5C), with notable variations in the proportions of macrophages, NK cells, fibroblasts, and endothelial cells. The cell-type composition of each sample is presented in Figure 5D. Figure 5E illustrates the differential expression of SGPP2 across various cell types in the IHF cohort in comparison to the none cohort. In the IHF group, SGPP2 expression was markedly decreased in fibroblasts, while a non-significant increase was observed in monocytes, T cells, and NK cells. These results demonstrate that downregulation of SGPP2 is closely associated with myocardial fibrosis and immune infiltration in IHF.

### 3.6. The Results of Immune Infiltration Analysis

**Figure 6:**
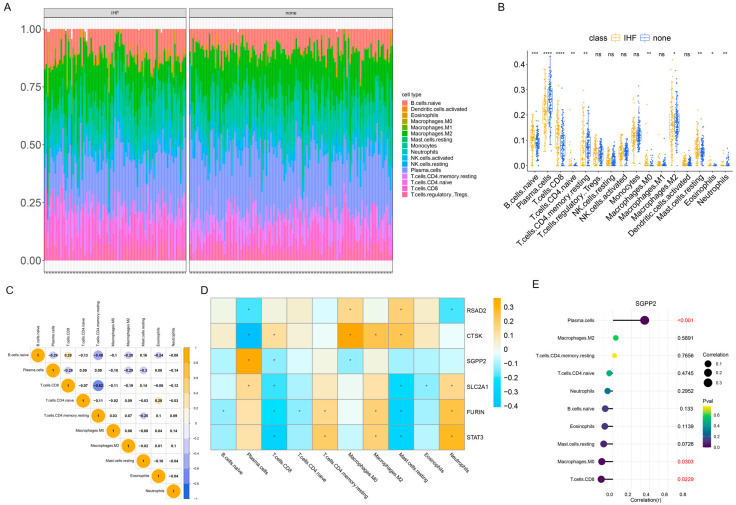
Immune infiltration analysis. (**A**): Proportions of different cell types in each sample. (**B**): Immune infiltration scores of different cell types in the two groups. (**C**): Correlations among the 10 cell types with significantly different immune infiltration scores. (**D**): Correlations between the 6 key genes and the 10 significantly different cell types. (**E**): Correlations between SGPP2 and the 10 significantly different cell types. * *p* < 0.05, ** *p* < 0.01, *** *p* < 0.001, **** *p* < 0.0001, ns represents unlimited statistical difference.

The relative proportions of cell types in each sample (Figure 6A) were analyzed via CIBERSORT. Compared to none, the IHF group showed lower infiltration scores for M2 macrophage, plasma, and resting memory CD4^+^T cells, but higher scores for naïve B, CD8^+^T, naïve CD4^+^T, M0 macrophage, resting mast cells, eosinophils, and neutrophils (Figure 6B). These findings suggest that the IHF microenvironment is characterized by a strong immune and inflammatory response. Correlations among cell types with significant infiltration differences are shown in Figure 6C, while associations between the six key genes and the ten differential cell types are presented in Figure 6D. Notably, SGPP2 negatively correlated with resting memory CD4^+^T cells and M2 macrophages, but positively with plasma cells (Figure 6E). These results suggest that SGPP2 may regulate an immunosuppressive or tissue repair-focused microenvironment, support the maintenance of long-term immune memory, and suppress plasma cell activity.

### 3.7. SGEA

**Figure 7:**
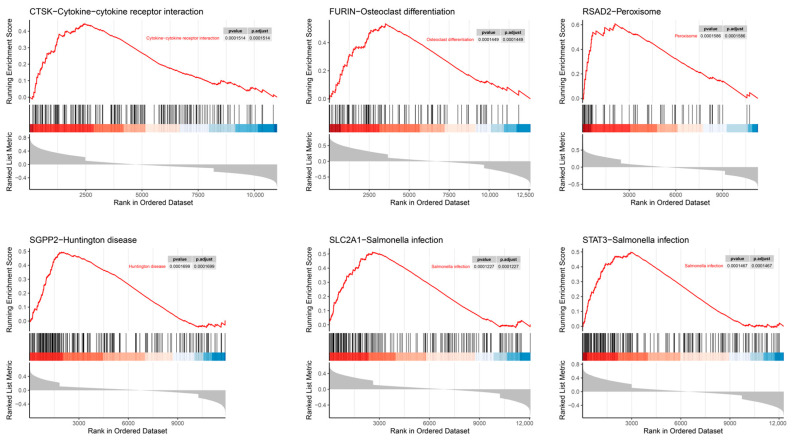
SGEA.

Figure 7 displays the top KEGG pathway of the six key genes, with all significantly associated signaling pathways detailed in Supplementary File S2. Notably, SGPP2 was significantly linked to the AMPK signaling pathway, which can regulate ERS and protect against myocardial injury, thereby ameliorating HF [7,8]. SIRT1, a key signaling factor in the AMPK pathway, also modulates ERS [15,16], and the SIRT1/AMPK signaling pathway is essential for preserving cardiac function in HF rats [17]. Based on these insights, the present study focuses on the SIRT1/AMPK signaling pathway as a core mechanism to further investigate the role of SGPP2 in IHF.

### 3.8. SGPP2 Is Downregulated in Rats with IHF

**Figure 8:**
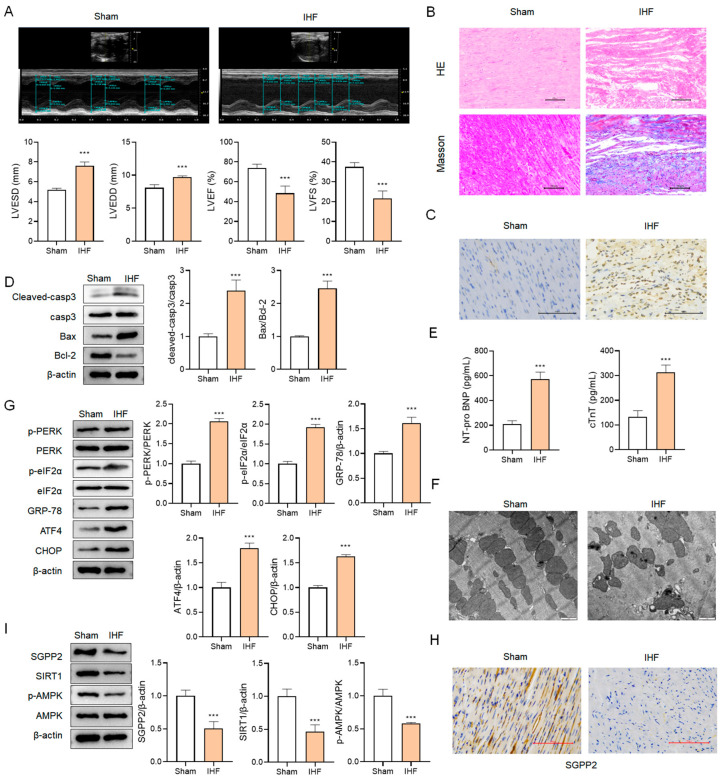
SGPP2 is downregulated in rats with IHF. (**A**): Echocardiography for assessment of cardiac function. (**B**): HE and Masson staining for myocardial histopathological evaluation. (**C**): TUNEL staining for the visualization of cardiomyocyte apoptosis. (**D**): WB analysis of apoptosis-linked proteins (casp3, Cleaved-casp3, Bax, and Bcl-2) in myocardial tissue. (**E**): Measurement of serum NT-proBNP and cTnT levels via ELISA. (**F**): Observation of endoplasmic reticulum via TEM. (**G**): WB analysis of ERS-linked proteins (PERK, p-PERK, eIF2α, p-eIF2α, GRP-78, ATF4, and CHOP) in myocardial tissue. (**H**,**I**): IHC and WB analysis of SGPP2 protein expression and SIRT1/AMPK signaling pathway activity in myocardial tissue. *** *p* < 0.001 vs. the Sham group; *n* = 6.

In comparison to the Sham group, rats with IHF exhibited significantly increased LVESD and LVEDD (*p* < 0.001), and markedly decreased LVEF and LVFS (*p* < 0.001) (Figure 8A). Myocardial tissue displayed disordered cellular structure, loose arrangement, marked immune infiltration, abundant blue collagen deposition, and apoptotic cells (Figure 8B,C). Additionally, increased Cleaved-casp3 expression and Bax/Bcl-2 ratio in myocardial tissue and elevated serum levels of NT-proBNP and cTnT were observed (all *p* < 0.001, Figure 8D,E). These results demonstrate that rats with IHF exhibit significantly impaired cardiac function, myocardial injury, and marked fibrosis. As shown in Figure 8F,G, rats with IHF exhibited swollen, vacuolated, and fragmented endoplasmic reticulum in cardiomyocytes, along with significantly increased expression of p-PERK, p-eIF2α, GRP-78, ATF4, and CHOP proteins (*p* < 0.001), indicating severe ERS. Furthermore, protein levels of SGPP2, SIRT1, and p-AMPK were markedly reduced in rats with IHF (Figure 8H,I, *p* < 0.001), suggesting downregulation of SGPP2 and decreased activity of the SIRT1/AMPK signaling pathway.

### 3.9. SGPP2 Alleviates OGD-Triggered Cardiomyocyte Injury

**Figure 9:**
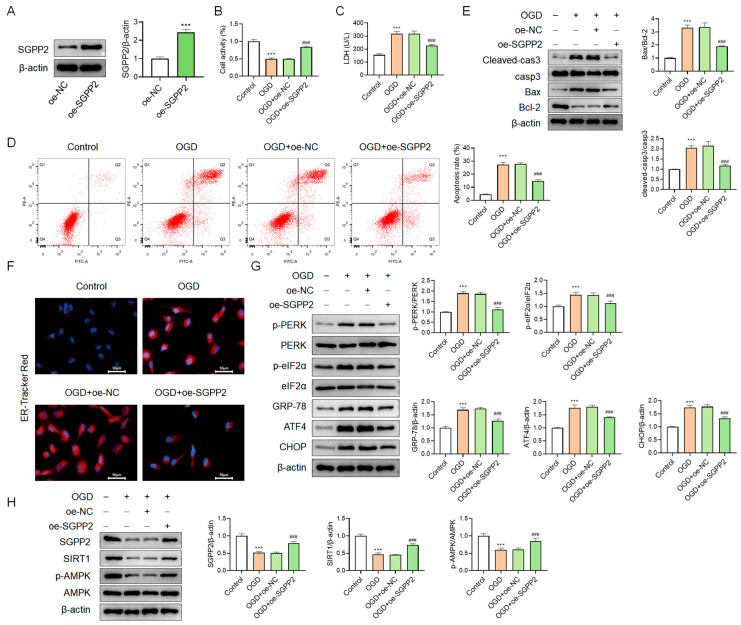
SGPP2 alleviates OGD-triggered cardiomyocyte injury. (**A**): WB validation of SGPP2 overexpression. (**B**): CCK-8 assay for cell viability detection. (**C**): LDH release assay for the evaluation of cell injury. (**D**,**E**): Flow cytometry and WB for the assessment of apoptosis in NRCMs. (**F**): Labeling of endoplasmic reticulum using the ER-Tracker Red molecular probe. (**G**,**H**): WB analysis of ERS-linked proteins, SGPP2, SIRT1, AMPK, and p-AMPK. *** *p* < 0.001 vs. the oe-NC or the Control group; ^###^ *p* < 0.001 vs. the OGD+oe-NC group; *n* = 3.

To investigate the role of SGPP2, SGPP2-overexpressing NRCMs were established (Figure 9A). In comparison to the Control group, the OGD group displayed lower cell viability (*p* < 0.001, Figure 9B), increased LDH release (*p* < 0.001, Figure 9C), and elevated apoptosis rate (*p* < 0.001, Figure 9D), as well as significantly elevated Cleaved-casp3 protein expression and Bax/Bcl-2 ratio (*p* < 0.001, Figure 9E). In contrast, the OGD+oe-SGPP2 group showed significantly ameliorated cell injury compared with the OGD+oe-NC group. These results demonstrate that SGPP2 alleviates OGD-induced injury in NRCMs. In addition, relative to the Control group, the OGD group displayed increased ER-Tracker Red fluorescence intensity (Figure 9F), and significantly elevated protein expression of p-PERK, p-eIF2α, GRP-78, ATF4, and CHOP (*p* < 0.001, Figure 9G), while the expression of SGPP2, SIRT1, and p-AMPK was notably reduced (*p* < 0.001, Figure 9H). In comparison to the OGD+oe-NC cohort, the OGD+oe-SGPP2 group displayed significant suppression of ERS-related indicators, along with markedly increased expression of SGPP2, SIRT1, and p-AMPK (*p* < 0.001). These findings indicate that SGPP2 significantly inhibits OGD-induced ERS. Furthermore, in OGD-induced NRCMs, SGPP2 overexpression significantly enhanced the activity of the SIRT1/AMPK signaling pathway.

### 3.10. SGPP2 Alleviates OGD-Triggered Cardiomyocyte Injury by Modulating ERS

**Figure 10:**
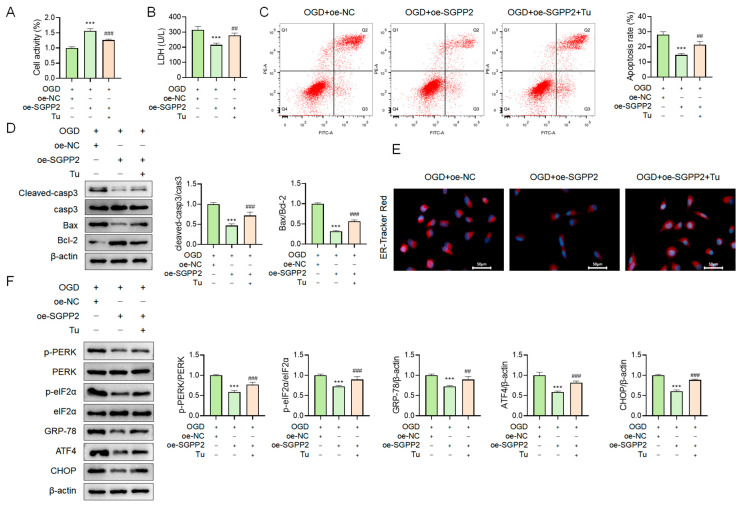
SGPP2 alleviates OGD-triggered cardiomyocyte injury by modulating ERS. (**A**): CCK-8 assay for cell viability detection. (**B**): LDH release assay for cell injury assessment. (**C**,**D**): Flow cytometry and WB for the assessment of apoptosis in NRCMs. (**E**): Labeling of endoplasmic reticulum using the ER-Tracker Red molecular probe. (**F**): WB analysis of ERS-related protein expression. *** *p* < 0.001 vs. the OGD+oe-NC group; ^##^ *p* < 0.01, ^###^ *p* < 0.001 vs. the OGD+oe-SGPP2 group; *n* = 3.

Consistent with the results shown in Figure 9, overexpression of SGPP2 significantly alleviated OGD-induced cardiomyocyte injury and suppressed ERS. Notably, relative to the OGD+oe-SGPP2 group, the OGD+oe-SGPP2+Tu group exhibited reduced cell viability (*p* < 0.001, Figure 10A), increased LDH release (*p* < 0.01, Figure 10B), elevated apoptosis rate (*p* < 0.01, Figure 10C), increased Cleaved-casp3 protein expression and Bax/Bcl-2 ratio (*p* < 0.001, Figure 10D), enhanced ER-Tracker Red fluorescence intensity (Figure 10E), and significantly increased protein expression of p-PERK, p-eIF2α, GRP-78, ATF4, as well as CHOP (*p* < 0.001, Figure 10F). These results demonstrate that SGPP2 alleviates OGD-induced cardiomyocyte injury by inhibiting ERS.

### 3.11. SGPP2 Alleviates OGD-Induced Cardiomyocyte ERS via the SIRT1/AMPK Signaling Pathway

**Figure 11:**
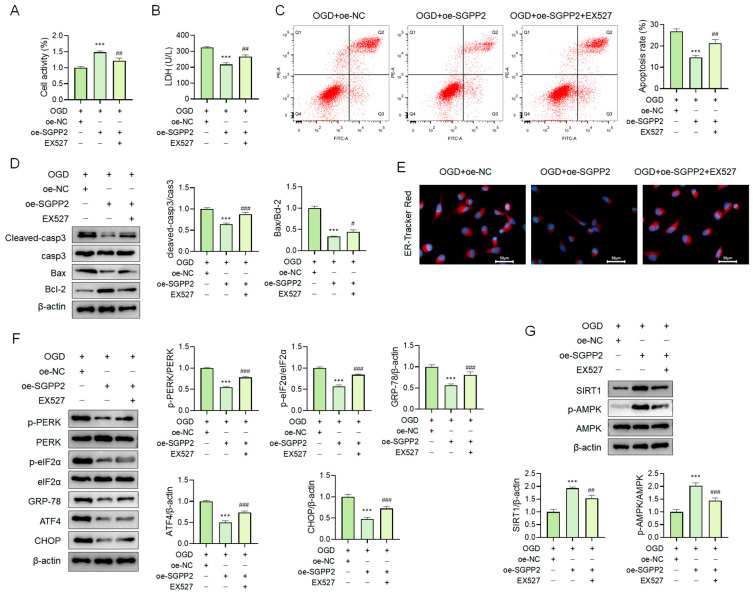
SGPP2 alleviates OGD-induced cardiomyocyte ERS via the SIRT1/AMPK signaling pathway. (**A**): CCK-8 assay for the detection of cell viability. (**B**): LDH release assay for the evaluation of cell injury. (**C**,**D**): Flow cytometry and WB for the assessment of apoptosis in NRCMs. (**E**): Labeling of endoplasmic reticulum using the ER-Tracker Red molecular probe. (**F**,**G**): WB analysis of the expression of ERS-related proteins and the activity of the SIRT1/AMPK signaling pathway. *** *p* < 0.001 vs. the 0.001 vs. the OGD+oe-NC group; ^#^ *p* < 0.05, ^##^ *p* < 0.01, ^###^ *p* < 0.001 vs. the OGD+oe-SGPP2 group; *n* = 3.

Consistent with the results in Figure 9, overexpression of SGPP2 alleviated OGD-triggered cardiomyocyte injury by suppressing ERS and upregulating the activity of the SIRT1/AMPK signaling pathway. Notably, in comparison to the OGD+oe-SGPP2 group, the OGD+oe-SGPP2+EX527 group exhibited decreased cell viability (Figure 11A), increased LDH release (Figure 11B), elevated apoptosis rate (all *p* < 0.01, Figure 11C), significantly increased Cleaved-casp3 protein expression and Bax/Bcl-2 ratio (*p* < 0.05, Figure 11D), enhanced ER-Tracker Red fluorescence intensity (Figure 11E), increased protein expression of p-PERK, p-eIF2α, GRP-78, ATF4, and CHOP, and significantly reduced SIRT1/AMPK signaling pathway activity (*p* < 0.01, Figure 11F,G). Therefore, SGPP2 inhibits ERS by activating the SIRT1/AMPK signaling pathway, thus alleviating OGD-induced cardiomyocyte injury.

## 4. Discussion

ERS is critical in the progression of various cardiovascular diseases like myocardial hypertrophy, ischemic heart disease, and HF [3,18]. Increasing evidence indicates that ERS is not only a key pathophysiological process in HF occurrence and development but also a potential therapeutic target for intervention [19]. For instance, Sabirli et al. reported elevated serum PERK levels in HF patients and observed a significant correlation between hospitalization duration and serum CHOP concentration in HF patients with reduced LVEF [20]. Similarly, clinical research by Momot et al. demonstrated that ERS is more pronounced in HF patients with reduced LVEF compared to those with preserved LVEF [21]. In a pentobarbital sodium-induced rabbit model of acute HF, Apelin ameliorated cardiac damage caused by acute HF by suppressing ERS [22]. Consistent with these findings, our study revealed a marked increase in ERS in myocardial tissues of IHF rats and in OGD-induced NRCMs. Moreover, ERS inducers markedly promoted OGD-induced ERS and injury in NRCMs. Collectively, these results demonstrate that targeting ERS represents a promising strategy for ameliorating HF.

ERS-related genes were identified via bioinformatics analysis, among which SGPP2 was found to be downregulated in IHF. In both in vivo and in vitro experiments, SGPP2 protein expression was significantly decreased in myocardial tissues of IHF rats and in OGD-induced NRCMs. Consistently, Wang et al. also reported downregulated expression of SGPP2 in HF [23]. SGPP2, a key enzyme in sphingolipid metabolism, negatively regulates S1P signaling by dephosphorylating S1P to sphingosine, and its dysregulated expression has been associated with various diseases [24,25,26,27]. In our in vitro experiments, SGPP2 overexpression was found to significantly inhibit OGD-induced ERS and ameliorate cellular injury. These findings align with those of Taguchi et al., who demonstrated that SGPP2-deficient cells exhibited increased ERS [12].

Our KEGG pathway enrichment analysis revealed a significant relation of SGPP2 to the AMPK signaling pathway. AMPK is a well-established energy sensor, and its activation shifts cellular metabolism from synthesis to catabolism [15]. AMPK is a widely recognized key therapeutic target for HF [15]. Reported evidence indicates that the cardioprotection of the AMPK signal in the myocardium involves many mechanisms [28]. Activation of AMPK mediates energy replenishment and reduces inflammation, thereby conferring cardiovascular benefits in patients with HF [7]. Furthermore, Chen et al. demonstrated that β-hydroxybutyrate inhibits ERS-mediated apoptosis via the AMPK pathway, thereby alleviating atherosclerotic calcification [29]. Collectively, these findings suggest that targeting ERS through the AMPK pathway may represent a promising therapeutic strategy for HF.

SIRT1 exerts protective effects against inflammation, vascular aging, atherosclerosis, and heart disease [30]. SIRT1 expression is downregulated in rat models of HF, whereas SIRT1 overexpression improves cardiac function and inhibits cardiomyocyte apoptosis [31]. Moreover, SIRT1 mitigates injury in the liver, heart, lungs, kidneys, and intestines by suppressing ERS [16]. Importantly, SIRT1 functions as a key regulator of the AMPK signaling pathway [15]. Yu et al. reported that Guiling Shugan Decoction enhances myocardial antioxidant capacity and preserves mitochondrial function, thereby improving cardiac function in HF rats through activating the SIRT1/AMPK signaling pathway [17]. Activation of this pathway has also been shown to inhibit ERS [9]. Consistent with these findings, our in vitro experiments demonstrated that SGPP2 overexpression significantly enhanced the activity of the SIRT1/AMPK signaling pathway and ameliorated OGD-induced ERS and cellular injury, whereas treatment with the SIRT1 inhibitor EX527 markedly suppressed the effects caused by SGPP2 overexpression.

Although the OGD-based cardiomyocyte model reproduces cellular ischemic stress, it cannot adequately capture the in vivo complexity of chronic IHF, including its structural, hemodynamic, and multicellular dimensions. Our in vivo observations consistently connect SGPP2 downregulation with IHF-related cardiac dysfunction. However, a direct demonstration of the functional effects of altering SGPP2 expression in vivo is still absent. Hence, it must be stressed that SGPP2 should currently be regarded as a mechanistically implicated candidate regulator of ERS and myocardial injury in IHF, not as an unequivocally confirmed therapeutic target.

## 5. Conclusions

This study unravels the mechanism through which SGPP2 ameliorates IHF by modulating ERS, thereby advancing our understanding of the pathogenesis of IHF and highlighting ERS as a promising therapeutic target. Furthermore, SGPP2 emerges as a promising candidate for intervention in IHF. However, the foregoing findings are primarily based on in vitro experiments, and the cardioprotective effects and underlying mechanisms of SGPP2 require further validation in vivo. Future studies integrating gene therapy and multi-omics approaches are warranted to confirm the therapeutic potential of SGPP2 in preclinical models and clinical settings.

## Data Availability

The original contributions presented in this study are included in the article/Supplementary Materials. Further inquiries can be directed to the corresponding author.

## Supplementary Materials

The following supporting information can be downloaded at: https://www.mdpi.com/article/10.3390/cimb48010100/s1.

## Abbreviations

The following abbreviations are used in this manuscript:

SGPP2	sphingosine-1-phosphatase 2
ERS	endoplasmic reticulum stress
SIRT1	sirtuin 1
AMPK	AMP-activated protein kinase
HF	heart failure
ICM	ischemic cardiomyopathy
IHF	ischemic cardiomyopathy-induced chronic heart failure
NRCMs	neonatal rat cardiomyocytes
OGD	oxygen-glucose deprivation
Tu	tunicamycin
UPR	unfolded protein response
PERK	protein kinase R-like endoplasmic reticulum kinase
ATF	activating transcription factor
CHOP	C/EBP-homologous protein
S1P	sphingosine-1-phosphate
DEGs	differentially expressed genes
GSVA	gene set variation analysis
WGCNA	weighted gene co-expression network analysis
PPI	protein–protein interaction
GO	Gene Ontology
KEGG	Kyoto Encyclopedia of Genes and Genomes
BP	biological process
CC	cellular component
MF	molecular function
SGEA	single-gene enrichment analysis
SD	Sprague–Dawley
LVEF	left ventricular ejection fraction
LVEDD	left ventricular end-diastolic diameter
LVEDS	left ventricular end-systolic diameter
LVFS	left ventricular fractional shortening
NT-proBNP	N-terminal Pro-B-type Natriuretic Peptide
OD	optical density
IHC	immunohistochemistry
DMEM	Dulbecco’s modified Eagle medium
oe-SGPP2	SGPP2 overexpression plasmid
oe-NC	negative control plasmid
CCK-8	Cell Counting Kit-8
LDH	Lactate Dehydrogenase
TEM	Transmission Electron Microscopy
WB	Western Blot
