# Supplementary material for: SGPP2 Ameliorates Chronic Heart Failure by Attenuating ERS via the SIRT1/AMPK Pathway
Source: Curr Issues Mol Biol. 2026 Jan 19;48(1):100. doi: 10.3390/cimb48010100 (PMC12840272; doi:10.3390/cimb48010100)
Supplement: Supplementary file 1 [file cimb-48-00100-s001.zip › Supplementary Figure S1.pdf]

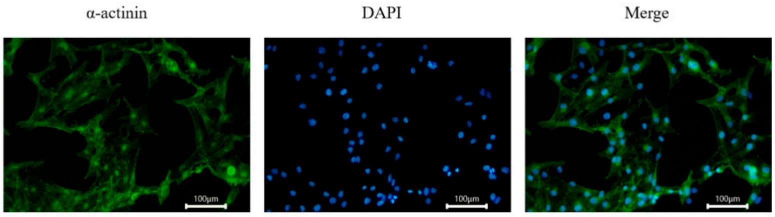

Supplementary Figure S1: Identification of NRCMs by immunofluorescence (α-actinin+DAPI). The cells in the image are extended, spindle-shaped or irregularly polygonal, with parallel striations (green) filling the cytoplasm and oval nuclei (blue) located centrally or peripherally.
